# Pyroptosis-Related Gene Signature Predicts the Prognosis and Immune Infiltration in Neuroblastoma

**DOI:** 10.3389/fgene.2022.809587

**Published:** 2022-05-19

**Authors:** Wanrong Li, Xin Li, Yuren Xia, Jian Wang

**Affiliations:** ^1^ Tianjin Cancer Hospital Airport Hospital, Tianjin, China; ^2^ Tianjin Medical University Cancer Institute and Hospital, National Clinical Research Center for Cancer, Key Laboratory of Cancer Prevention and Therapy, Tianjin’s Clinical Research Center for Cancer, Tianjin, China

**Keywords:** neuroblastoma, pyroptosis, prognostic model, bioinformatics, overall survival

## Abstract

Neuroblastoma is the most common pediatric extracranial solid tumor. The 5-year survival rate for high-risk neuroblastoma is less than 50%, despite multimodal treatment. Pyroptosis, an inflammatory type of programmed cell death, manifested pro-tumor and anti-tumor roles in the adult tumor. Thus, we aimed to elucidate the function of pyroptosis in neuroblastoma. We classified neuroblastoma patients into two clusters based on the pyroptosis gene expression. We found high pyroptosis neuroblastoma manifested favorable overall survival and more anti-tumor immune cell infiltration. Based on the results of a stepwise Cox regression analysis, we built a four-gene predictive model including NLRP3, CASP3, IL18, and GSDMB. The model showed excellent predictive performance in internal and external validation. Our findings highlight that high pyroptosis positively correlated with neuroblastoma outcomes and immune landscape, which may pave the way for further studies on inducing pyroptosis therapy in high-risk neuroblastoma treatment.

## Introduction

Neuroblastoma is a heterogeneous pediatric tumor, in which clinical behavior changes from spontaneous regression to widespread metastasis (Maris, 2010; Huang and Weiss, 2013). Low-risk neuroblastoma has 90%–95% survival rates for 5 years. However, despite intensive multimodal therapy, the high-risk neuroblastoma has less than 50% survival rates for 5 years (Swift et al., 2018). Thus, new therapy options for high-risk neuroblastoma are urgently needed.

Pyroptosis is an inflammatory type of programmed cell death, which requires gasdermin proteins for plasma membrane perforation, often due to inflammatory caspase activation (Broz et al., 2020). Recent evidence suggests pyroptosis performs dual roles on the adult tumor. On the one hand, based on the theories of inflammation-cancer transformation and chronic inflammation-induced cell carcinogenesis, pyroptosis, as an inflammatory process, forms a suitable microenvironment for cervical and colorectal cancer growth. On the other hand, inducing pyroptosis activates an immune response in the tumor microenvironment, turning up the heat on non-immunoreactive tumors such as triple-negative breast cancer, and suppressing tumor proliferation (Xia et al., 2019; Wu et al., 2021; Yu et al., 2021). Pyroptosis exert different function in different tumors. Neuroblastoma is immunologically “cold” since lacking anti-tumor T-cell infiltration and a low mutation burden (Wienke et al., 2021). However, the specific function of pyroptosis in neuroblastoma has been less studied.

Herein, we aim to elucidate the role of pyroptosis in neuroblastoma. We investigate the correlation between pyroptosis signature, overall neuroblastoma survival, and immune microenvironment through comprehensive bioinformatics analyses. It is hoped that this research will contribute to a deeper understanding of pyroptosis in the neuroblastoma immune landscape and treatment.

## Materials and Methods

### Acquisition of Gene Expression and Clinical Data

Neuroblastoma gene expression datasets and related clinical data were downloaded from the Gene Expression Omnibus (GEO) under accession number GSE49710 and ArrayExpress under accession number E-MTAB-8248. The GSE49710 datasets were used to construct the predictive model. The E-MTAB-8248 datasets were used to validate the predictive model.

The count matrix of the fetal adrenal gland and fetal adrenal medulla single-cell datasets were downloaded from the Shiny App. The count matrix was processed and annotated following the author protocol (Jansky et al., 2021).

### Consensus Clustering

Pyroptosis-related genes were extracted from previous articles (Shi et al., 2016; Xia et al., 2019; Liu X et al., 2021) and listed in Supplementary Table S1. The expression data of pyroptosis-related genes were extracted from the GSE49710 dataset. The data were normalized by median value before clustering. The “ConsensusClusterPlus” package was used to cluster samples (Wilkerson and Hayes, 2010).

### Differentially Expressed Gene Identification and Integrated Analysis

Patients were stratified into different groups according to the consensus cluster results. Probes were matched to the gene symbols using the annotation files provided by the manufacturer. The highest expression value was employed to represent the gene expression level when a single gene matched multiple probes. Differentially expressed genes (DEGs) were explored between groups using the limma package (Ritchie et al., 2015). The DEG cutoff were set as |log_2_ (fold-change) | > 1 and adjusted *p-*value < 0.05.

### Bioinformatic and Protein–Protein Interaction Analysis of Differentially Expressed Genes

GO enrichment and KEGG pathway analyses were used to explore the potential biological processes (BP), cellular components (CC), and molecular functions (MF) of DEGs in the ClusterProfiler package (Yu et al., 2012). ***p*** < 0.05 was considered statistically significant. Gene set enrichment analysis (GSEA) was performed to elucidate the molecular mechanisms of DEGs. |NES| > 1 and FDR < 0.05 were considered statistically significant.

### Tumor Immunity Analyses

Stromal, immune, and estimate scores were calculated by the Estimation of STromal and Immune cells in MAlignant Tumor tissues using the Expression data (ESTIMATE) algorithm in the IOBR package (Yoshihara et al., 2013; Zeng et al., 2021). Eight immune cells were scored by the Microenvironment Cell Populations-Counter (MCP-counter) algorithm in the IOBR package (Racle et al., 2017; Zeng et al., 2021). A proportion of twenty-two immune cells were evaluated by the Cell Type Identification by Estimating Relative Subsets of RNA Transcripts (CIBERSORT) algorithm in the IOBR package (Newman et al., 2015; Zeng et al., 2021).

### Identification of Survival-Related Pyroptosis Genes and Establishment of Prognostic Gene Signature

Stepwise Cox regression analysis was used to identify overall survival-related genes in the GSE49710 dataset. The pyroptosis-related genes were considered statistically significant where *p* < 0.01 in the univariate Cox regression analysis and included in subsequent analysis. Least absolute shrinkage and selection operator (LASSO)-penalized cox regression analysis was performed to further reduce the number of DEGs with the best predictive performance using 10-fold cross-validation in the glmnet package (Friedman et al., 2010). The multivariate Cox regression analysis was used to optimize the DEGs under the minimum AIC value. A prognostic signature was constructed based on the linear combination of the regression coefficients (β) derived from the multivariate cox regression model multiplied by its mRNA expression level. Patients were divided into high-risk and low-risk groups based on the median risk value. Kaplan–Meier analysis, the area under the curve (AUC) of the receiver operating characteristic (ROC) curve, and Harrell’s concordance index (C-index) were used to evaluate the performance of the prognostic signature.

The E-MTAB-8248 dataset was used for the prognostic signature validation. The risk score was calculated by the same formula used for the GSE49710 dataset. The patients in the E-MTAB-8248 cohort were divided into low-risk or high-risk groups by the median risk score calculated from the GSE49710 dataset. These groups were then compared to validate the prognostic model.

### Identification of Independent Prognostic Parameters of Neuroblastoma

In order to identify independent prognostic parameters, a univariate Cox regression analysis was performed based on the prognostic gene signature and clinic-pathological parameters, including age, sex, INSS stage, and MYCN status. *p* < 0.05 was considered statistically significant.

### Predictive Nomogram Construction and Validation

After testing collinearity, all independent prognostic parameters were included in constructing a predictive nomogram to predict a 1-, 3-, and 5-year overall survival of neuroblastoma patients in the GSE49710 dataset. The Kaplan–Meier analysis, the AUC of the ROC curve, Harrell’s concordance index, and a calibration plot were used to evaluate the performance of the prognostic nomogram. The patients were divided into two groups based on the median points of the nomogram. Survival curves for two groups were plotted using the Kaplan–Meier analysis.

### Statistical Analysis

Statistical analysis was performed in R. The overall survival between subgroups was compared by the Kaplan–Meier method. The Cox regression analysis was performed to evaluate the overall survival-related parameters. The Mann–Whitney test was used to compare immune cell infiltration between subgroups. Unless otherwise stated, ***p*** < 0.05 was considered statistically significant.

## Results

### Neuroblastoma Classification Based on Pyroptosis-Related Genes

**FIGURE 1:**
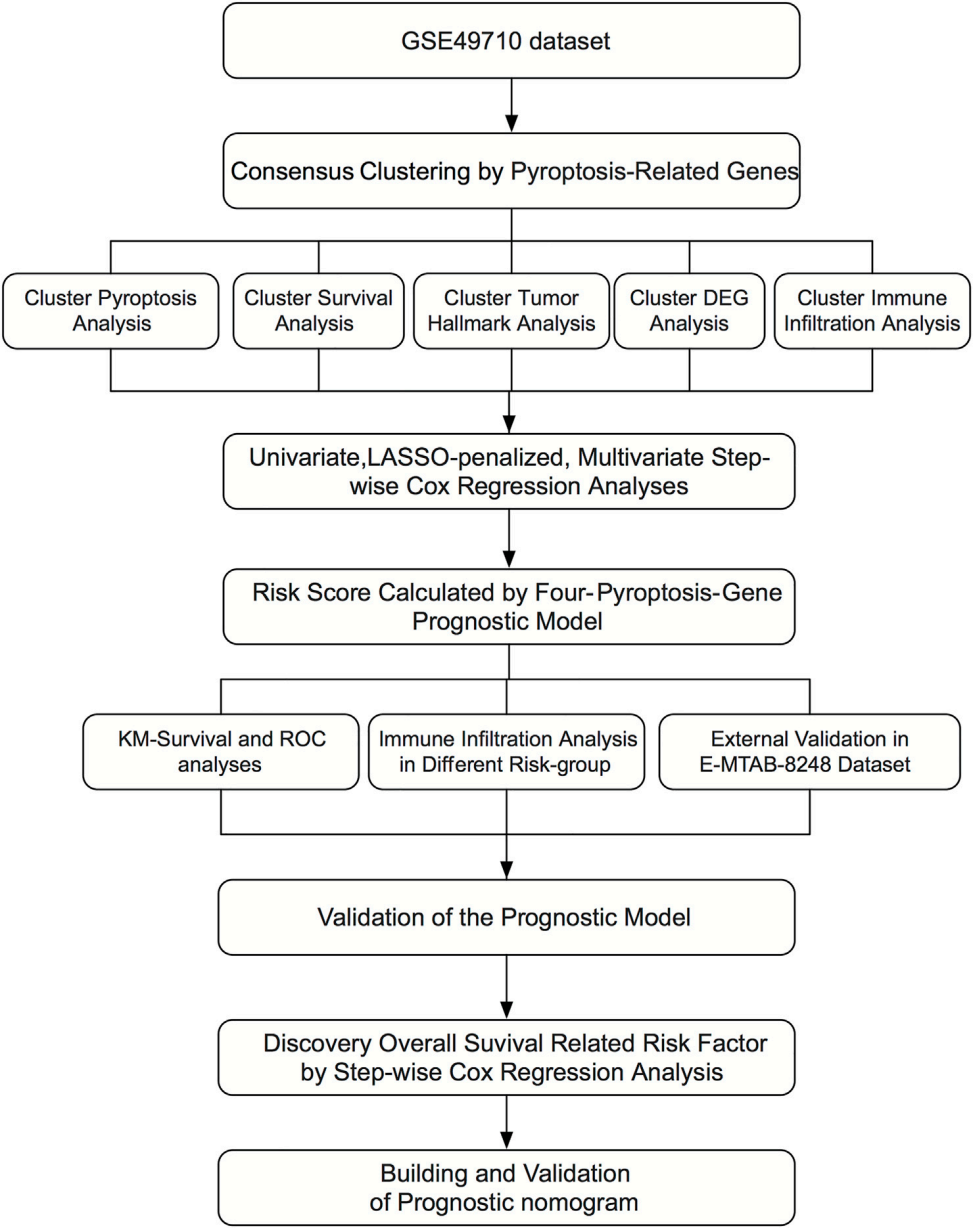
Diagram illustrating the research design.

**FIGURE 2:**
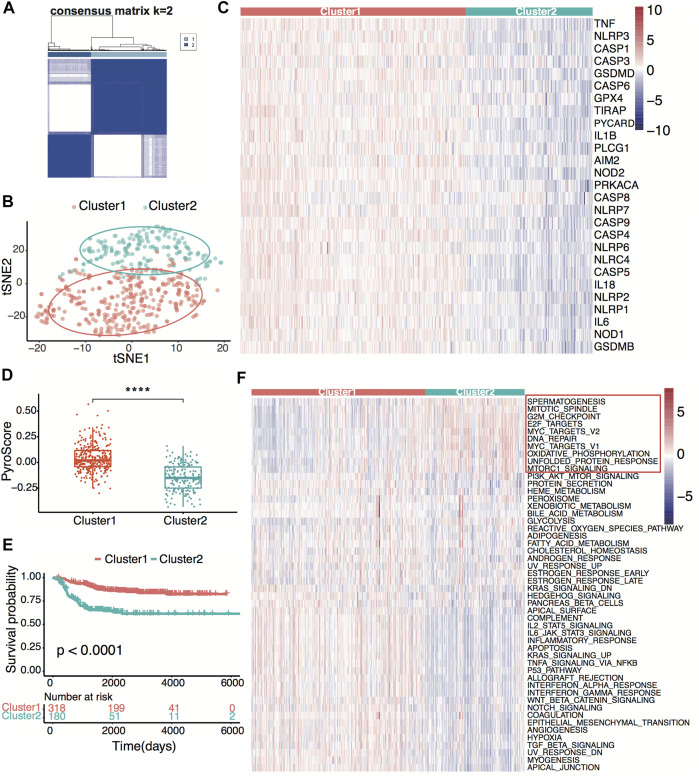
Neuroblastoma classification based on pyroptosis-related genes. **(A)** Consensus clustering for 498 neuroblastoma patients in the GSE49710 dataset at *k* = 2. **(B)** *t*-distributed stochastic neighbor embedding (t-SNE) visualization of the two clusters. **(C)** Heatmap showing expression of the pyroptosis-related genes in the two clusters. **(D)** Box plots of the distribution of the pyroptosis signature score calculated by the ssGSEA algorithm between cluster 1 and cluster 2. ****p < 0.0001, two-sided unpaired Wilcoxon test. **(E)** Kaplan–Meier curves for overall survival of the two clusters. The *p*-value was calculated by the log-rank test. **(F)** Heatmap showing scores of the tumor hallmark calculated by the ssGSEA algorithm in the two clusters. Ten high hallmarks in cluster 2 are highlighted.

Figure 1 shows the flowchart of this research. A total of 27 of 33 pre-defined pyroptosis-related genes were found in the GSE49710 dataset. We clustered 498 tumor samples by the 27 pyroptosis-related genes to explore the internal connection. By applying the clustering variable (*k*) from 2 to 6, we found *k* = 2 performance satisfying. Intragroup correlations were high, and intergroup correlations were low. Based on the clustering results, 498 neuroblastoma samples were divided into two clusters, while cluster 1 contains 318 samples and cluster 2 contains 180 samples (Figure 2A). Two clusters exhibited considerable separation (Figure 2B). Compared with cluster 2, cluster 1 showed a higher pyroptosis-related gene expression (Figure 2C). To quantify the expression level in two clusters, we calculated the pyroptosis signature score by the ssGSEA algorithm (Zeng et al., 2021). Cluster 1 showed a significantly higher pyro-score than cluster 2 (Figure 2D). The Kaplan–Meier survival curves revealed significantly favorable overall survival in cluster 1 (Figure 2E, ***p*** < 0.0001). Furthermore, we scored the cancer hallmark signature by the ssGSEA algorithm to compare the tumor hallmark difference between clusters. Compared with cluster 1, cluster 2 showed a higher score in cell cycle, DNA repair, and MYC target signature (Figure 2F), indicating cell replication and MYC-related pathway may play an important role in the neuroblastoma outcome.

### Cluster Character Exploration

**FIGURE 3:**
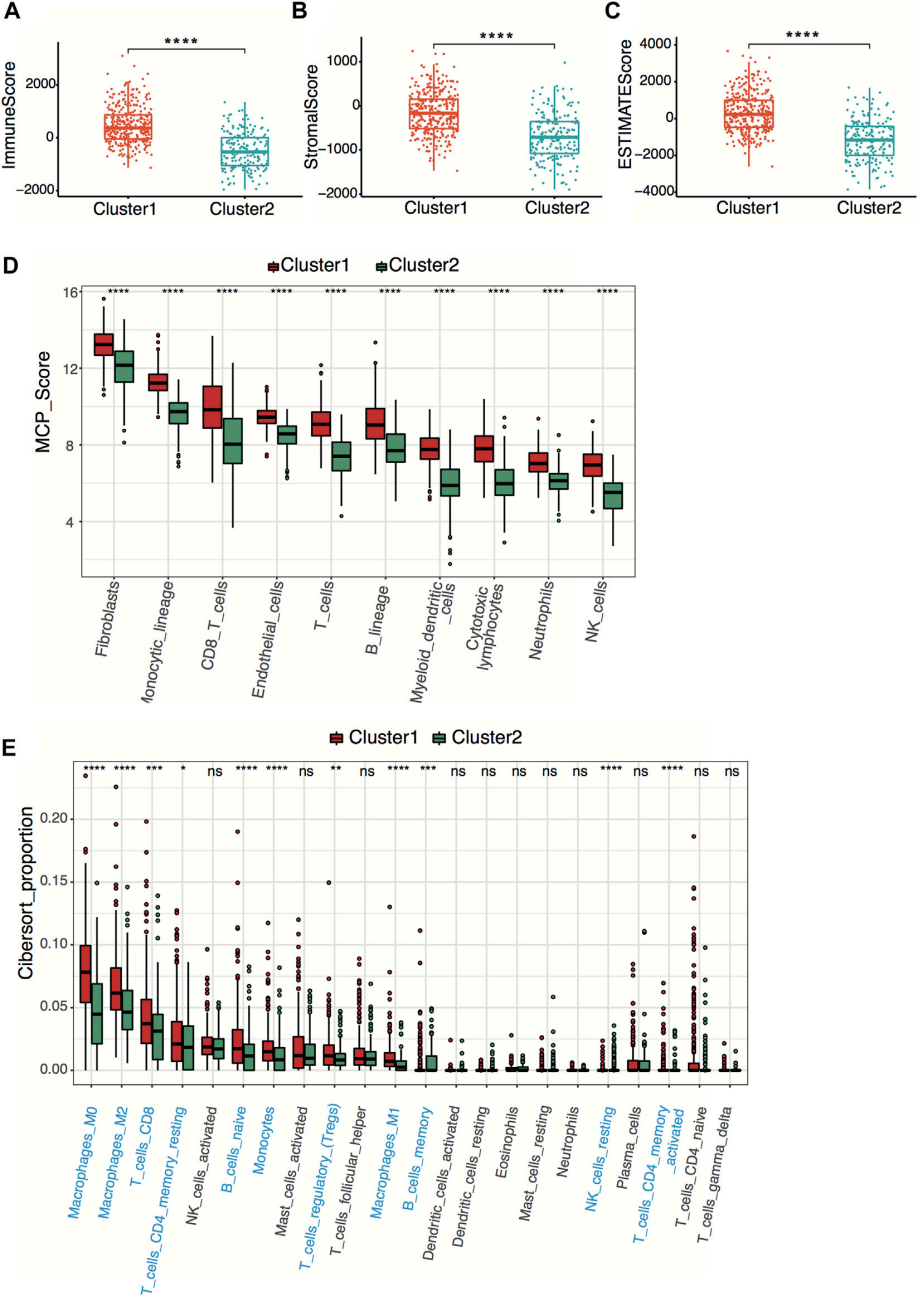
Neuroblastoma immunity analysis between clusters. **(A–C)** Box plots of the distribution of the immune score **(A)**, stromal score **(B)**, and estimate score **(C)** calculated by the ESTIMATE algorithm between cluster 1 and cluster 2. ****p < 0.0001, two-sided unpaired Wilcoxon test. **(D)** Box plots of the distribution of the scores calculated by the MCP-counter algorithm of immune cells between cluster 1 and cluster 2. ****p < 0.0001, two-sided unpaired Wilcoxon test. **(E)** Box plots of the distribution of the cell proportions calculated by the CIBERSORT algorithm of immune cells between cluster 1 and cluster 2. Statistically different immune cells are highlighted in blue. *p < 0.05, **p < 0.01, ***p < 0.001, ****p < 0.0001, n.s., not significant, two-sided unpaired Wilcoxon test.

To explore tumor immunity difference between clusters, we first evaluated the stromal cell and immune cell infiltration in tumor tissues using the ESTIMATE algorithm. Compared with cluster 2, cluster 1 showed significantly higher stromal, estimate, and immune scores, indicating more immune-cell and stromal-cell infiltration in cluster 1 (Figures 3A–C). To further elucidate the tumor-infiltrating immune cells, the MCP-counter and CIBERSORT algorithms were applied. Anti-tumor immune cells, including cytotoxic lymphocytes, macrophage, and natural killer cells, infiltrated higher in cluster 1 (Figures 3D,E), implying higher pyroptosis expression may induce more anti-tumor immune cell infiltration and activate the immune response in the tumor microenvironment.

To further identify the pyroptosis signature-related pathway, we analyzed the differential expression genes (DEGs) between two clusters, with the criteria set as |logFC|>1 and adjusted *p*-value < 0.05. Compared with cluster 2, we identified 3876 upregulated and 11 downregulated genes in cluster 1 (Supplementary Figure S1A). Interestingly, the 11 downregulated genes contain the MYCN gene, which is frequently used in clinical prognosis prediction (Supplementary Figure S1A). GO, KEGG, and GSEA pathway enrichment analyses were applied to discover the functions of the DEGs. The DEGs were significantly enriched in biological processes related to immune cell activation and immune response, consistent with the regulating cell death and inflammation character of pyroptosis. Enrichment analyses of the cellular compartment were mainly on plasma membrane complex and extracellular matrix, corresponding to the immune-complex cellular location (Supplementary Figure S1B). KEGG pathway analysis revealed that the DEGs participated in cytokine receptor interaction, immune cell cytotoxicity, cell adhesion molecules, and glucose metabolism (Supplementary Figure S1C). Furthermore, the GSEA analysis revealed NF-κB signaling pathway and antigen processing involvement in the pyroptosis process (Supplementary Figure S1D).

### Identification of Survival-Related Pyroptosis Signature Genes and Establishment of Pyroptosis Four-Gene Prognostic Signature

**FIGURE 4:**
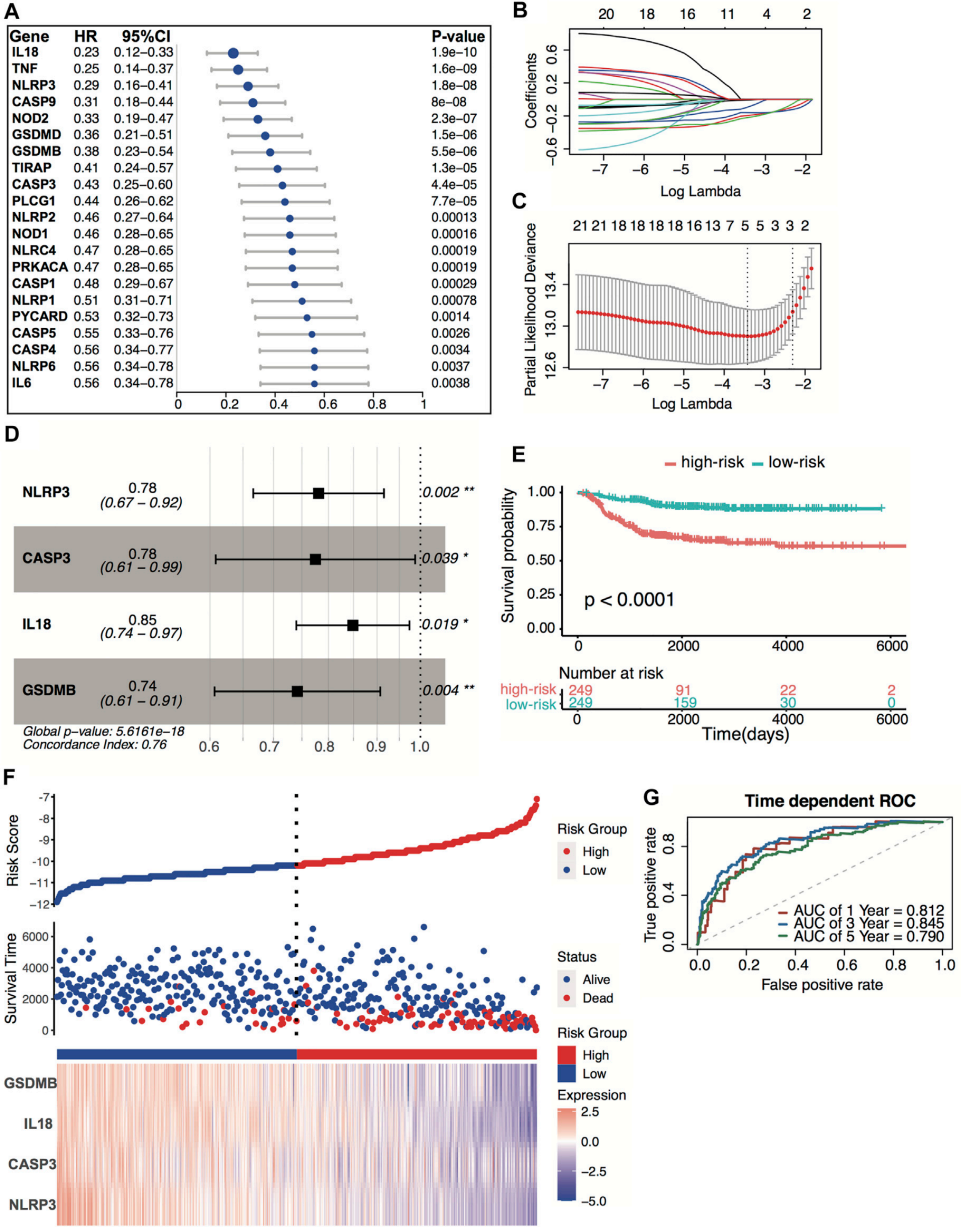
Construction of the predictive four-pyroptosis-gene signature. **(A)** Prognostic effect of 21 pyroptosis-related genes with *p* < 0.01 derived from univariate Cox regression survival analyses for overall survival in the GSE49710 dataset. **(B)** The LASSO coefficient profile of 21 pyroptosis-related genes in the GSE49710 dataset. **(C)** The 10-fold cross-validation for tuning predictor selection. **(D)** Prognostic effect of four-gene signature derived from a stepwise Cox regression survival analysis for overall survival in the GSE49710 dataset. *p < 0.05, **p < 0.01. **(E)** Kaplan–Meier curves for overall survival of the two risk groups derived from the four-gene signature in the GSE49710 dataset. The *p*-value was calculated by the log-rank test. **(F)** Distribution of the risk score, the associated survival data, and the four-gene mRNA expression in the GSE49710 dataset. **(G)** ROC curves for 1-, 3-, and 5-year overall survival predictions for the four-gene signature in the GSE49710 dataset.

All 498 patients from the GSE49710 dataset with sufficient survival information were included in subsequent survival analyses. Supplementary Table S2 shows the clinical information of these patients. Based on the univariate Cox regression analysis, we identified 21 pyroptosis genes, which were significantly associated with overall survival. The hazard ratio of 21 genes was less than 1, indicating that pyroptosis signature might serve as a protective factor in neuroblastoma (Figure 4A). The Kaplan–Meier survival revealed significantly favorable overall survival of all the 21 genes (Supplementary Table S3). A prognostic signature comprising four genes, including NLR family pyrin domain containing 3 (NLRP3), caspase-3 (CASP3), interleukin 18 (IL18), gasdermin B (GSDMB), was developed by LASSO-penalized Cox and multivariate Cox analyses (Figures 4B–D). The risk score was calculated by the following equation:

Risk score = [(−0.24719) × expression value of NLRP3] + [(−0.25486) × expression value of CASP3] + [(−0.16392) × expression value of IL18] + [(−0.29864) × expression value of GSDMB]

The median risk score (−10.67) was set as the cutoff value. Patients from the GSE49710 dataset were stratified into two groups. The Kaplan–Meier survival curves revealed significantly favorable overall survival in the groups with lower risk scores (Figures 4E–F, *p* < 0.0001). Time-dependent ROC and C-index were applied to determine the prognostic values of the four-gene model. The AUCs for 1-, 3-, and 5-year overall survival predictions for the model were 0.812, 0.845, and 0.790, respectively (Figure 4G). The C-index of the four-gene model was 0.763 (95% CI: 0.720–0.806), indicating that the four-gene signature performed well at predicting the overall survival of neuroblastoma.

### External Validation of the Four-Gene Signature

**FIGURE 5:**
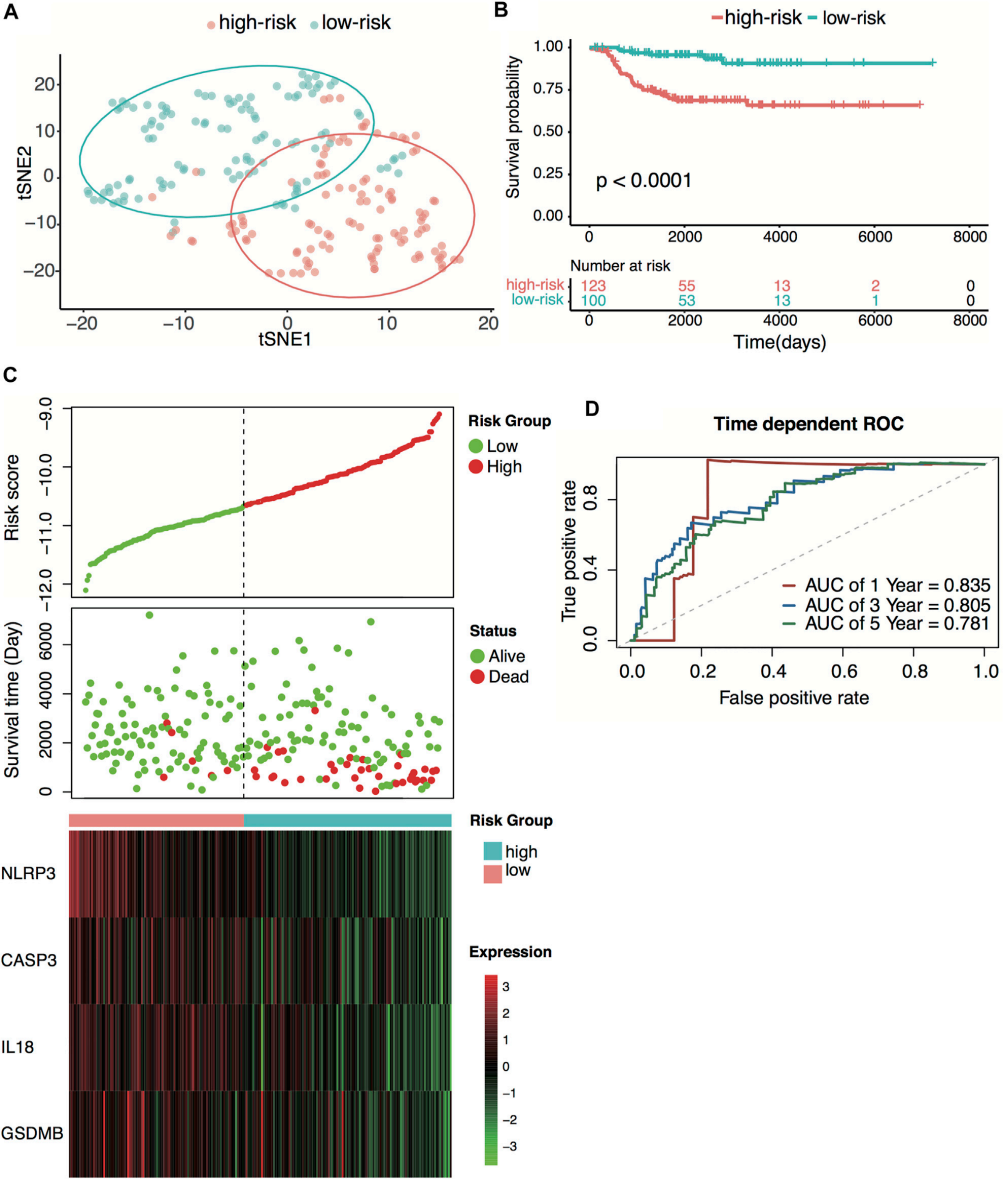
External validation of the four-pyroptosis-gene signature. **(A)** t-SNE visualization of the two risk groups derived from the four-gene signature in the E-MTAB-8248 dataset. **(B)** Kaplan–Meier curves for overall survival of the two risk groups derived from the four-gene signature in the E-MTAB-8248 dataset. The *p*-value was calculated by the log-rank test. **(C)** Distribution of the risk score, the associated survival data, and the four-gene mRNA expression in the E-MTAB-8248 dataset. **(D)** Receiver operating characteristic (ROC) curves for 1-, 3-, and 5-year overall survival predictions for the four-gene signature in the E-MTAB-8248 dataset.

E-MTAB-8248 dataset was used to validate the prediction performance of the four-gene prognostic signature. The risk score was calculated with the same formula for each patient. Patients were divided into high- and low-risk groups according to the median risk score (−10.67) calculated from the GSE49710 dataset. Two groups exhibited considerable separation through t-SNE analysis (Figure 5A). The Kaplan–Meier survival curve revealed a significant difference in overall survival between groups. High-risk groups had markedly poorer outcomes than low-risk groups (Figures 5B,C). Predictive power was then assessed by time-dependent ROC and C-index. In the E-MTAB-8248 dataset, the AUCs for 1-, 3-, and 5-year overall survival predictions for the risk scores were 0.835, 0.805, and 0.781, respectively (Figure 5D). The C-index of the risk score was 0.764 (95% CI: 0.703–0.825). External validation indicated that the four-gene signature performed well at predicting overall survival in neuroblastoma patients.

### Single Gene Survival Analysis of the Four-Gene Signature

The GSE49710 and E-MTAB-8248 datasets were used to explore each gene expression’s significance on overall survival. Patients were divided into high-expression and low-expression groups according to the median expression value of each gene. The high-expression group of the four genes had markedly better outcomes than the low-expression group in both datasets, indicating that all four genes play a protection role in neuroblastoma (Supplementary Figures S2A,B).

### Clinical Pathology and Tumor Immunity Relevance of the Four-Gene Signature

**FIGURE 6:**
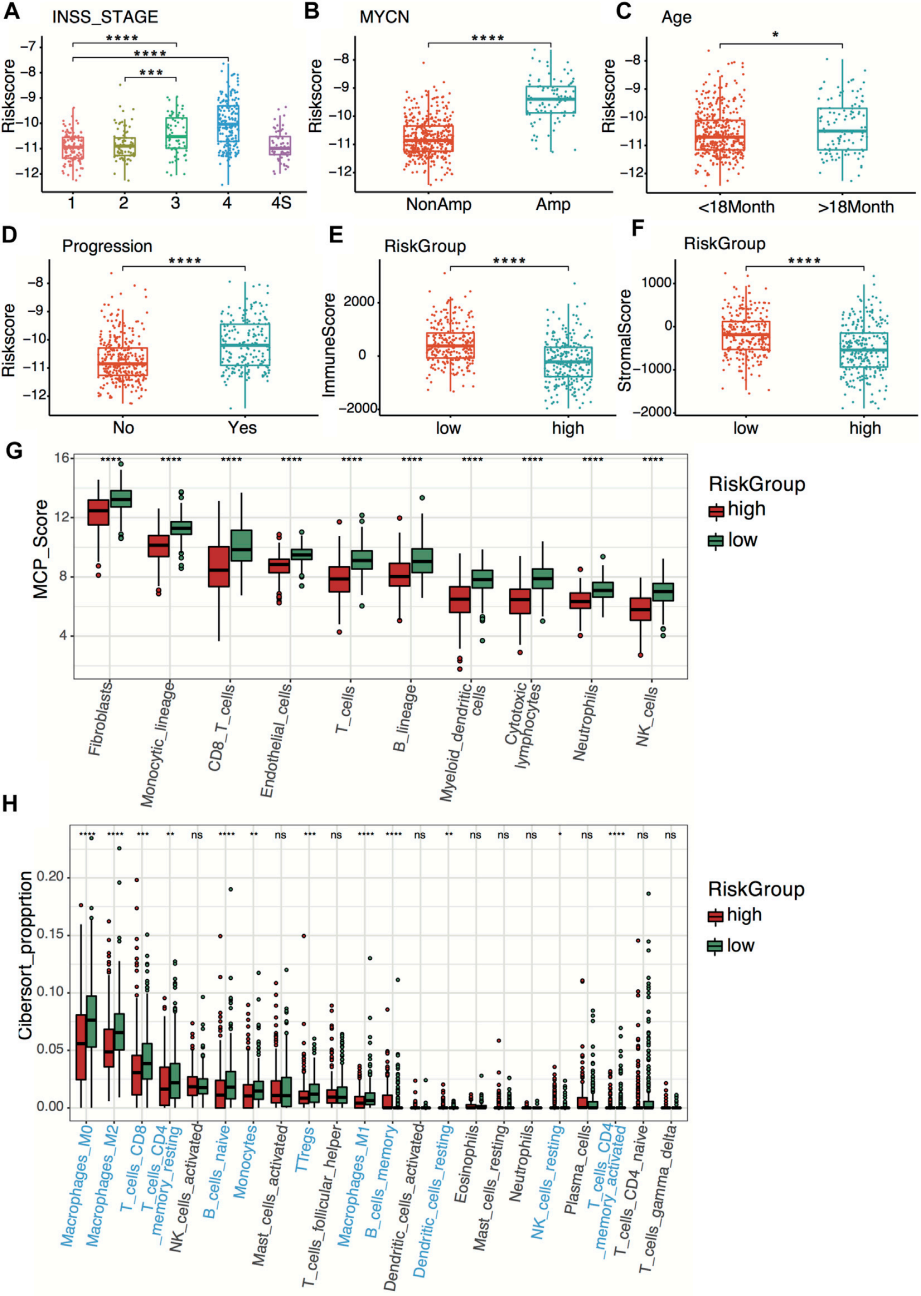
Clinical relevance and tumor immunity of the four-gene signature in the GSE49710 dataset. **(A–D)** Box plots of the distribution of the four-gene risk score between different INSS stage groups **(A)**, between the MYCN-amplified and non-MYCN-amplified groups **(B)**, between the age >18 months and age <18 months groups **(C)**, and between progression and non-progression groups **(D)**. *p < 0.05, ***p < 0.001, ****p < 0.0001, two-sided unpaired Wilcoxon test. **(E–F)** Box plots of the distribution of the immune score **(E)** and stromal score **(F)** calculated by the ESTIMATE algorithm between the high- and low-risk groups. ****p < 0.0001, two-sided unpaired Wilcoxon test. **(G)** Box plots of the distribution of the scores calculated by the MCP-counter algorithm of immune cells between the high-risk and low-risk groups. ****p < 0.0001, two-sided unpaired Wilcoxon test. **(H)** Box plots of the distribution of the cell proportions calculated by the CIBERSORT algorithm of immune cells between the high- and low-risk groups. Statistically different immune cells are highlighted in blue. *p < 0.05, **p < 0.01, ***p < 0.001, ****p < 0.0001, n.s., not significant, two-sided unpaired Wilcoxon test.

Relationships between the four-gene signature and the clinical characteristics of neuroblastoma, including the INSS (International Neuroblastoma Staging System) stage, age, MYCN status, and tumor progression, were analyzed in both datasets. In terms of INSS stage, stage III and stage IV patients had higher risk scores than stage I and stage II patients in both datasets (Figure 6A; Supplementary Figure S3A). The four-gene risk scores increased from stage 1 to stage 4 except for stage 4S. Patients of MYCN amplification and age >18 months had significantly higher four-gene risk scores in both datasets (Figures 6B,C; Supplementary Figures S3B,C). The GSE49710 dataset analysis revealed that patients with higher four-gene risk scores tended to have tumor progression (Figure 6D).

To explore tumor immunity relevance of the four-gene signature, we first evaluated the stromal cell and immune cell infiltration in tumor tissues using the ESTIMATE algorithm. Compared with the high-risk group, the low-risk group showed significantly higher stromal and immune scores in both datasets (Figures 6E,F; Supplementary Figures S3D,E), indicating more immune- and stromal-cell infiltration in the low-risk group. To further elucidate the tumor-infiltrating immune cells, the MCP-counter and CIBERSORT algorithms were applied. Anti-tumor immune cells, including cytotoxic lymphocytes, macrophage, and natural killer cells, infiltrated higher in the low-risk group (Figures 6G,H; Supplementary Figures S3F,G).

### Cellular Origin of the Four-Gene Signature

To explore the cellular origin of the four-pyroptosis-related genes, we projected the four genes separately onto the single-cell datasets of the fetal adrenal gland and fetal adrenal medulla by Jansky et al. (2021). We found that the NLRP3 and IL18 are mainly expressed in the immune cell, while the CASP3 and GSDMB are expressed in the fetal adrenal medulla, stromal, and immune cells (Supplementary Figures S4A). Further analysis found that CASP3 is mainly expressed on the neuroblasts, while the GSDMB is mainly expressed on the Schwann cell precursors (SCPs) of the fetal adrenal medulla (Supplementary Figures S4B).

### Model Comparison Between the Four-Gene Signature and the INSS Stage

**FIGURE 7:**
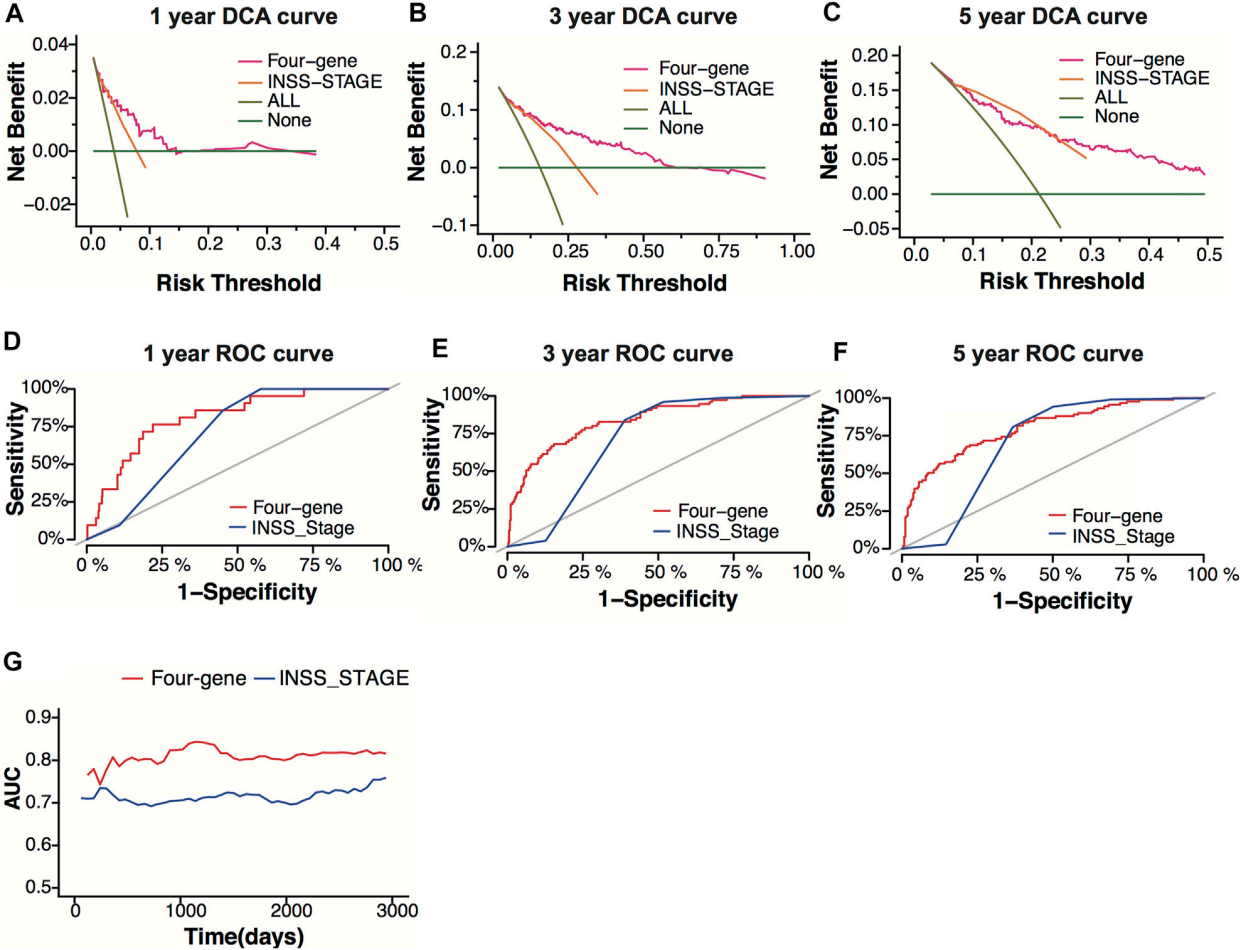
Model comparison between the four-gene signature and the INSS stage. **(A–C)** Decision-curve analysis (DCA) curves depicting the standardized net benefit of the four-gene signature and the INSS stage model for 1 **(A)**, 3 **(B)**, and 5 years **(C)** in the GSE49710 dataset. **(D–F)** ROC curves of the four-gene signature and the INSS stage model for 1- **(D)**, 3- **(E)**, and 5-year **(F)** overall survival predictions in the GSE49710 dataset. **(G)** The area under the curve (AUC) of the four-gene signature and the INSS stage model.

INSS stage is one of the systems used for neuroblastoma staging, which decides the risk group of neuroblastoma patients. To verify the prediction power of the four-gene signature, we compared the four-gene model with the INSS stage. The decision curve analysis (DCA) for 1-, 3-, and 5-year overall survival predictions showed better efficiency for the four-gene signature than the INSS stage (Figures 7A–C). The area under the decision curve was 0.0022, 0.034, and 0.045 of the four-gene signature for 1, 3, and 5 years, respectively. The area under the decision curve was 0.0012, 0.016, and 0.028 of the INSS stage for 1, 3, and 5 years, respectively. The ROC curve of the two models showed better prediction power for the four-gene signature (Figures 7D–F). The four-gene signature model showed a higher AUC value than the INSS stage model, whether short-term or long-term (Figure 7G).

### Evaluation of Prognostic Factors in Neuroblastoma and Building Nomogram Model

**FIGURE 8:**
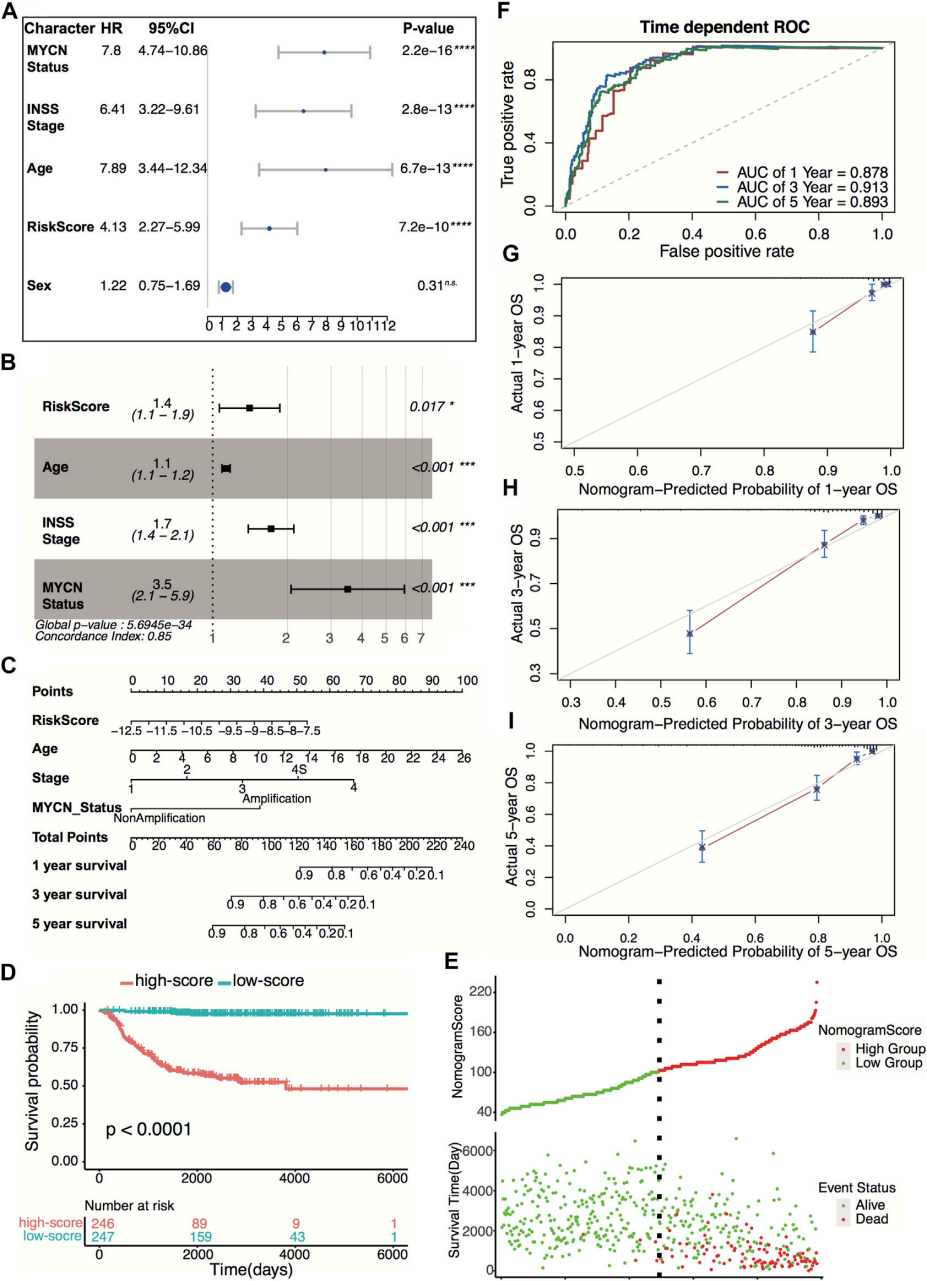
Construction and validation of the nomogram model. **(A)** Prognostic effect of MYCN status, INSS stage, age, four-gene risk score, and sex derived from univariate Cox regression survival analysis for overall survival in the GSE49710 dataset. ****p < 0.0001, n.s., not significant. **(B)** Prognostic effect of MYCN status, INSS stage, age, and four-gene risk score derived from multivariate Cox regression survival analysis for overall survival in the GSE49710 dataset. **(C)** The nomogram model for 1-, 3-, and 5-year overall survival probability predictions in the GSE49710 dataset. **(D)** Kaplan–Meier curves for overall survival of the two score groups derived from the nomogram model in the GSE49710 dataset. The *p*-value was calculated by the log-rank test. **(E)** Distribution of the nomogram score and the associated survival data in the GSE49710 dataset. **(F)** ROC curves for 1-, 3-, and 5-year overall survival predictions for the nomogram model in the GSE49710 dataset. **(G–I)** Calibration plots of 1, 3, and 5 years for internal validation of the nomogram model. The *y*-axis represents the actual overall survival, while the *x*-axis represents the predicted overall survival.

Four hundred ninety-three patients from the GSE49710 dataset, whose complete clinical information was provided, including age, sex, MYCN status, and INSS stage, were included in the analysis. Stepwise Cox regression analysis was used to identify overall survival related factors. The univariate Cox analysis revealed that the four-gene risk score, age, INSS stage, and MYCN status significantly correlated with overall survival (Figure 8A). Multivariate Cox analysis revealed that the four-gene risk score, age, INSS stage, and MYCN status were independent risk factors of overall survival (Figure 8B). A prognostic nomogram model was constructed based on the multivariate Cox regression results, predicting 1,- 3-, and 5-year overall survival (Figure 8C). The patients were divided into two groups by the scores of the nomogram. The Kaplan–Meier plot effectively discriminated groups of various risks. Higher scores had significantly poorer overall survival (Figures 8D,E, *p* < 0.0001). The AUCs of the 1-, 3-, and 5-year overall survival predictions for the nomogram model were 0.878, 0.913, and 0.893, respectively (Figure 8F). The C-index of the nomogram model was 0.851 (95% CI; 0.824–0.878). Calibration plots of 1, 3, and 5 years showed that the nomogram performed well at predicting overall survival in neuroblastoma patients (Figures 8G–I).

## Discussion

In the present study, we give a close correlation between pyroptosis signature, neuroblastoma immune landscape, and neuroblastoma outcomes. The high pyroptosis neuroblastoma manifested favorable overall survival, more CD8^+^ T cells, natural killer (NK) cells, and memory T-cell infiltration. In the adult tumor, previous studies found that pyroptotic tumor cells induce the activation of cytotoxic T cells and dendritic cells by releasing immunostimulatory cytokines (Shi et al., 2016; Wu et al., 2021). Less than 15% of breast tumor cell pyroptosis could eliminate the entire 4T1 tumor graft by activating cytotoxic T cells and CD4^+^ T helper cells (Wang et al., 2020). We found that the anti-tumor immune response plays a pivotal role in pyroptosis regulating neuroblastoma outcomes based on functional enrichment analyses. The main pathways involved in this process were antigen processing and NF-κB signaling pathway. MYCN amplification, the *de no* oncogene driver that accounts for 20% of neuroblastoma, is observed in high-risk neuroblastoma and poor patient survival (Otte et al., 2021). However, MYCN was considered undruggable since lacking a targetable surface on its DNA binding domain (Huang and Weiss, 2013; Liu Z et al., 2021). Surprisingly, we found that high pyroptosis neuroblastoma tends to have low expression of MYCN and a low score of myc target-related tumor hallmark. Further research on MYCN expression and pyroptosis signature may unravel new treatment options for high-risk neuroblastoma.

Based on stepwise Cox regression analysis, we constructed a four-gene signature containing NLRP3, CASP3, IL18, and GSDMB, which showed better predictive performance than conventional biomarkers. The four genes were protection factors in neuroblastoma since being downregulated is associated with poor survival. The four genes are distributed in all of the pyroptosis processes. The NLRP3 is an intracellular sensor that detects stimulations, resulting in the formation of the NLRP3 inflammasome (Swanson et al., 2019). The CASP3, the constituent of the NLRP3 inflammasome, is the activator that shears the gasdermin protein to induce pyroptosis (Tsuchiya, 2021). The GSDMB, one of the gasdermin superfamily, is the pyroptosis executor, which forms pores on the membrane (Li et al., 2020). The IL18, one of the pro-inflammatory cytokines, is the immune effector released from the pores formed by the gasdermin protein (Broz et al., 2020). The lack of NLRP3 significantly reduced lung cancer metastasis and improved melanoma survival rate while promoting colorectal cancer metastasis and hepatocellular carcinoma progression (Hamarsheh and Zeiser, 2020). Thus, NLRP3 plays different roles in various types of cancer. We found that high expression of NLRP3 was associated with a favorable outcome of neuroblastoma, which corresponds to the NLRP3 reducing the neuroblastoma SH-Y5Y cell line tumorsphere size (Tezcan et al., 2021). As the central caspases, CASP3 regulates apoptosis membrane blebbing and pyroptosis activation, controlling cell death fate (Tsuchiya, 2021). Higher levels of activated caspase-3 were correlated with increased tumor recurrence or death in the adult tumor (Huang et al., 2011). However, we found that high expression of caspase-3 was associated with a favorable outcome of neuroblastoma. The mechanism of caspase-3 regulation in neuroblastoma needs further research. GSDMB is the most divergent in the gasdermin superfamily, which is not present in the mouse and rat. GSDMB expression was increased in many cancers, such as breast, cervical, and hepatic cancer, in which high expression was linked to poor prognosis (Li et al., 2020). The role of GSDMB in neuroblastoma has been less studied. Contrary to the adult tumor, we found that higher GSDMB expression neuroblastoma manifested prolonged overall survival. Multiple neuroblastoma prognosis signatures have been made through various methods (Garcia et al., 2012; Zhong et al., 2018; Brady et al., 2020). These signatures showed excellent predictive performance of neuroblastoma outcomes while less correlated with the neuroblastoma immune landscape. The pyroptosis signature classified neuroblastoma patients with a significant difference in immune cell infiltration, providing new therapy options in neuroblastoma immunotherapy. We build a visualized scoring nomogram based on the pyroptosis signature and clinical-pathology characteristics. Through the nomogram, physicians can predict the overall survival and make treatment recommendations for each patient. Patients in the high-risk group should be given more attention and intensive treatment, while excessive treatment should be avoided for low-risk group patients.

Immunotherapy is a hotspot in cancer therapy. Since lacking anti-tumor T-cell infiltration and low mutation burden, the neuroblastoma is being immunologically “cold” (Wienke et al., 2021). The immune checkpoint blockade antibodies targeting CTLA4, PD-1, and PD-L1 have not influenced the clinical outcomes of neuroblastoma (Wienke et al., 2021). Dinutuximab, the GD2 antibody, was the FDA-approved immunotherapy for high-risk neuroblastoma. Dinutuximab significantly improved 2-year event-free survival but less contributed to the 5-year overall survival of the high-risk neuroblastoma (Mora, 2016). The biggest issue of dinutuximab could not induce immunological memory, which prevents high-risk neuroblastoma relapse (Szanto et al., 2020). Therefore, converting the immunosuppressive neuroblastoma into an immunostimulating environment and inducing immunological memory may be promising strategies. Pyroptosis, an inflammatory regulated cell death, showed excellent tumor elimination when combined with PD-1 antibody in the immunologically “cold” breast tumor grafts (Wang et al., 2020). A recent study found that pyroptosis turns bladder tumors from the “cold” to “hot” immune environment (Chen et al., 2021). In neuroblastoma, we found that high pyroptosis neuroblastoma tends to have more CD8^+^ T-cell, natural killer (NK) cell, and memory CD4^+^ T-cell infiltration, and favorable outcomes. The presence of natural killer cells in TME is associated with an improved prognosis of neuroblastoma. NK cells mediate cellular cytotoxicity, which is a mechanism of anti-GD2 for neuroblastoma, and a combination of anti-GD2 antibodies with adoptively transferred NK cells significantly improves neuroblastoma survival (Barry et al., 2019). Higher CD8^+^ T-cell abundance correlated with favorable prognosis and long-term survival of neuroblastoma (Mina et al., 2016). Memory T cells have been associated with favorable clinical outcomes in several solid tumors (Craig et al., 2020). Thus, compared with the low pyroptosis neuroblastoma, high pyroptosis neuroblastoma manifested the immunogenic “hot,” which means more anti-tumor immune cell infiltration. However, previous studies found that the anti-tumor immune cells in neuroblastoma showed low immune reactivity (Wienke et al., 2021). The reactivity of the anti-tumor immune cell induced by pyroptosis needs further research.

Our study found that the pyroptosis signature correlated with anti-tumor immune cell infiltration and neuroblastoma outcomes. However, several limitations exist in our research. First, pyroptosis showed both pro-tumor and anti-tumor effects in adult tumors; whether pyroptosis has a pro-tumor role and to what extent activates pyroptosis to induce tumor-killing without promoting tumor growth in neuroblastoma need to be studied further. Second, excerpting the anti-tumor immune cell infiltration, we also found the immunosuppressive Tregs infiltrated in the high pyroptosis neuroblastoma, and the balance between the anti-tumor immune cells and the immunosuppressive immune cells in neuroblastoma remains the further study. Third, our results are based on the bioinformatics analysis, which needs further experimental validation of the gene expression pattern.

## Conclusion

In conclusion, our finding highlighted that the high pyroptosis signature is positively correlated with anti-tumor immune cell infiltration and neuroblastoma patient outcomes. To the best of our knowledge, our study is the first bioinformatics analysis of the pyroptosis signature in neuroblastoma. Our findings pave the way for further studies on inducing pyroptosis therapy in high-risk neuroblastoma treatment.

## Data Availability

The datasets presented in this study can be found in online repositories. The names of the repository/repositories and accession number(s) can be found in the article/Supplementary Material.
