# Supplementary figures and images for: Pyroptosis-Related Gene Signature Predicts the Prognosis and Immune Infiltration in Neuroblastoma
Source: Front Genet. 2022 May 19;13:809587. doi: 10.3389/fgene.2022.809587 (PMC9162481; doi:10.3389/fgene.2022.809587)

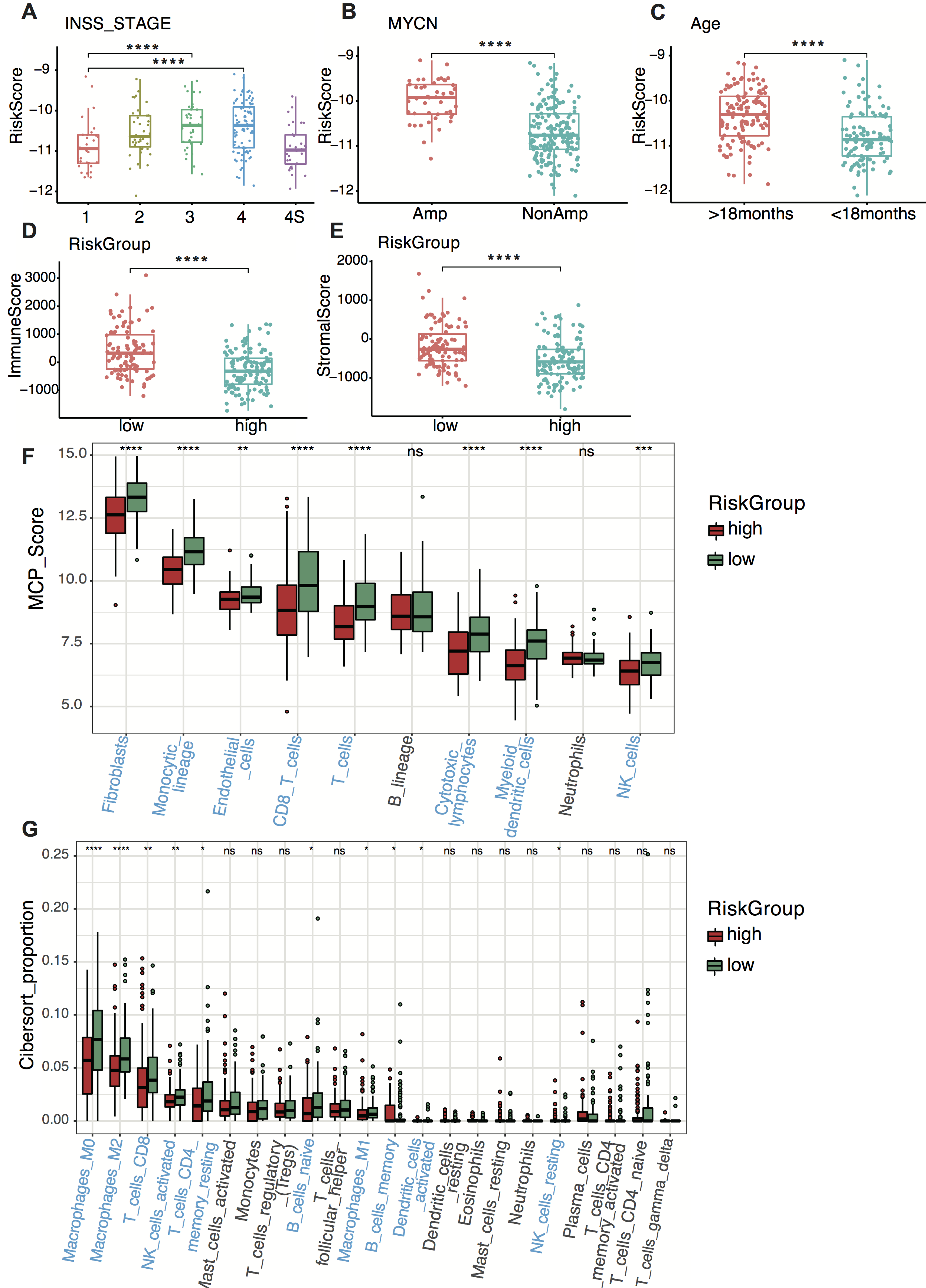

Supplement: Supplementary file 1 [file Image3.TIFF]

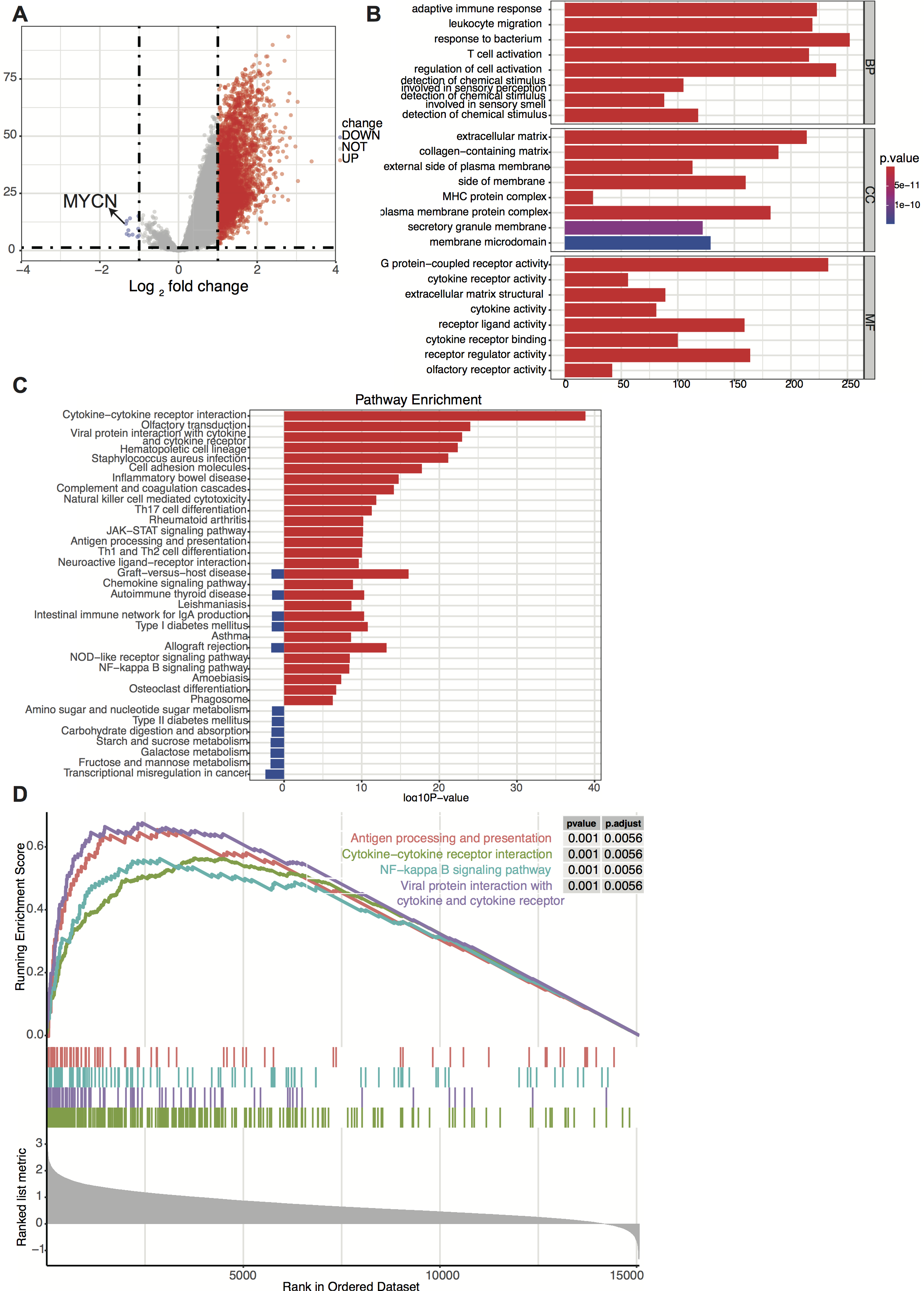

Supplement: Supplementary file 3 [file Image1.TIFF]

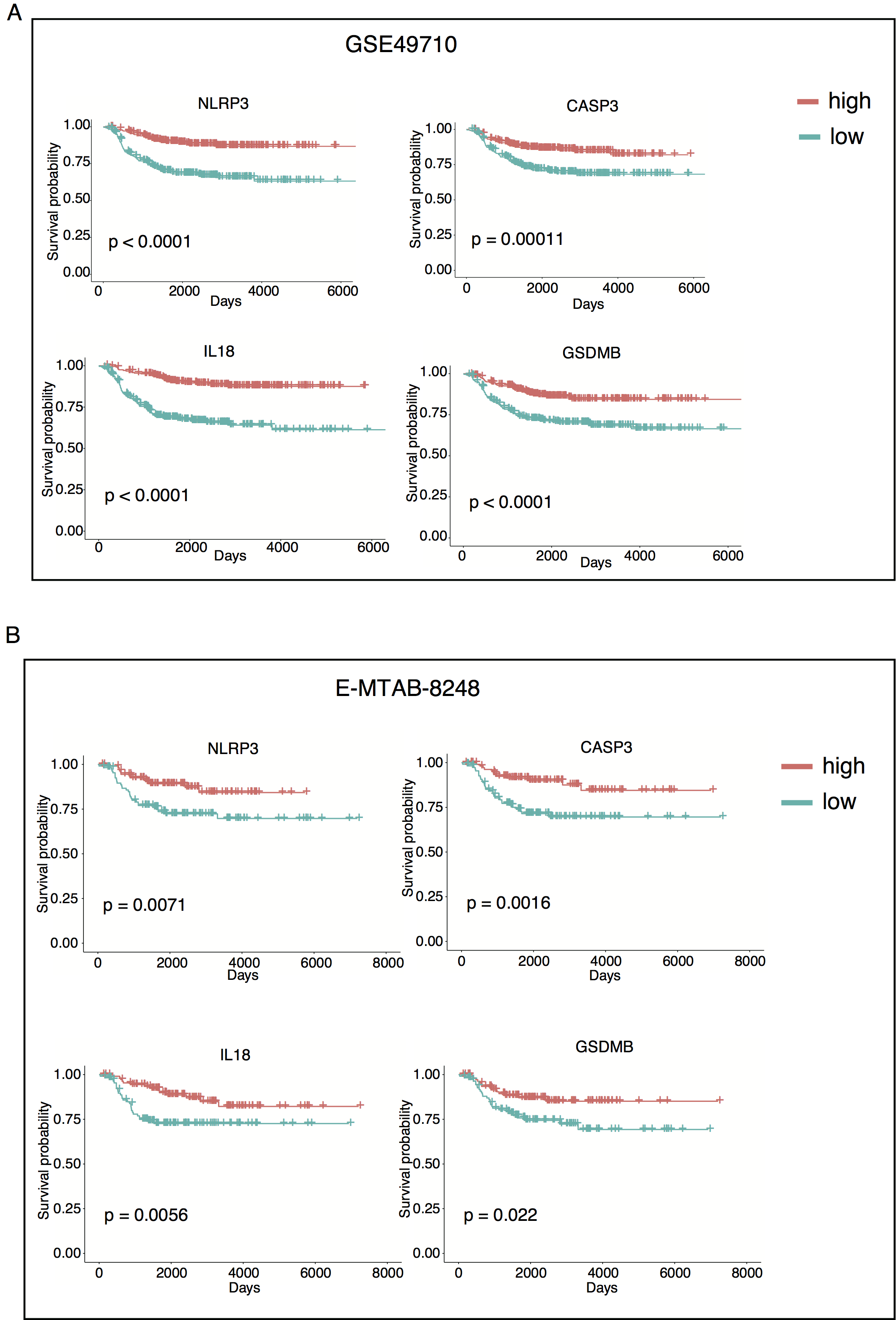

Supplement: Supplementary file 6 [file Image2.TIFF]

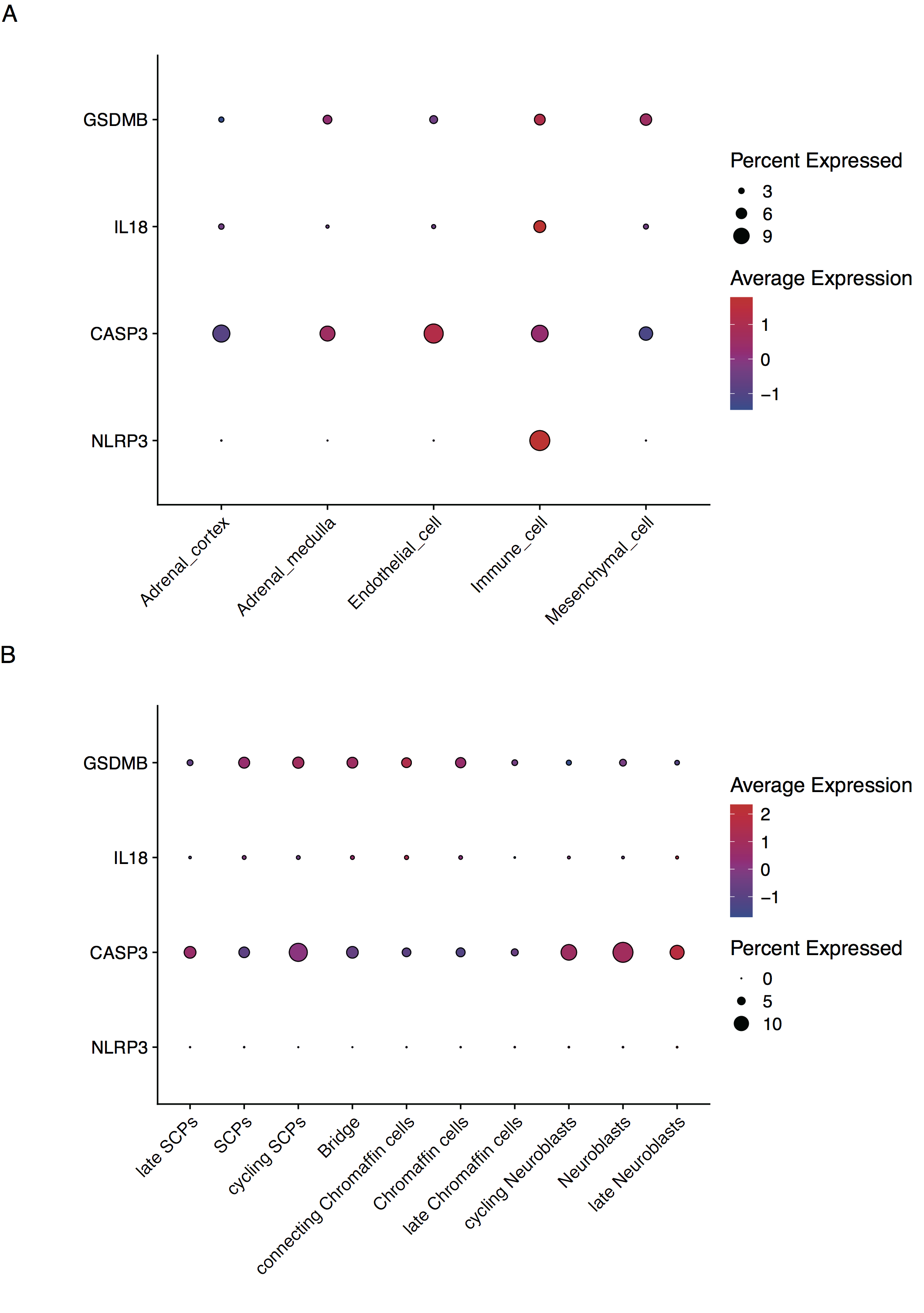

Supplement: Supplementary file 7 [file Image4.TIFF]
